# Characterization of the Cancer-Associated Meprin Βeta Variants G45R and G89R

**DOI:** 10.3389/fmolb.2021.702341

**Published:** 2021-10-06

**Authors:** Antonin Gellrich, Franka Scharfenberg, Florian Peters, Martin Sammel, Ole Helm, Fred Armbrust, Frederike Schmidt, Juliane Lokau, Christoph Garbers, Susanne Sebens, Philipp Arnold, Christoph Becker-Pauly

**Affiliations:** ^1^ Biochemical Institute, Kiel University, Kiel, Germany; ^2^ Department of Ophthalmology, Laboratory for Retinal Cell Biology, University Hospital Zurich, Zurich, Switzerland; ^3^ Institute for Experimental Cancer Research, Kiel University, Kiel, Germany; ^4^ Institute of Pathology, Otto-von-Guericke University Magdeburg, Magdeburg, Germany; ^5^ Institute of Functional and Clinical Anatomy, FAU Erlangen, Erlangen, Germany

**Keywords:** metalloprotease, meprin, sheddase, IL-6, CD99, APP, ADAM

## Abstract

Meprin *β* is a metalloprotease associated with neurodegeneration, inflammation, extracellular matrix homeostasis, transendothelial cell migration, and cancer. In this study, we investigated two melanoma-associated variants of meprin *β,* both exhibiting a single amino acid exchange, namely, meprin *β* G45R and G89R. Based on the structural data of meprin *β* and with regard to the position of the amino acid exchanges, we hypothesized an increase in proteolytic activity in the case of the G45R variant due to the induction of a potential new activation site and a decrease in proteolytic activity from the G89R variant due to structural instability. Indeed, the G89R variant showed, overall, a reduced expression level compared to wild-type meprin β, accompanied by decreased activity and lower cell surface expression but strong accumulation in the endoplasmic reticulum. This was further supported by the analysis of the shedding of the interleukin-6 receptor (IL-6R) by meprin *β* and its variants. In transfected HEK cells, the G89R variant was found to generate less soluble IL-6R, whereas the expression of meprin *β* G45R resulted in increased shedding of the IL-6R compared to wild-type meprin *β* and the G89R variant. A similar tendency of the induced shedding capacity of G45R was seen for the well-described meprin *β* substrate CD99. Furthermore, employing an assay for cell migration in a collagen IV matrix, we observed that the transfection of wild-type meprin *β* and the G45R variant resulted in increased migration of HeLa cells, while the G89R variant led to diminished mobility.

## Introduction

Meprin *β* is a metalloprotease of the astacin family of zinc endopeptidases. As a multidomain homodimer and a type I transmembrane protein, it is tethered to the cell surface or it can be shed from the plasma membrane by other proteases such as ADAMs (a disintegrin and metalloproteases) (Broder and Becker-Pauly 2013; Wichert et al., 2017; Scharfenberg et al., 2019). Expressed as a zymogen, meprin *β* must be activated by proteases with tryptic specificity due to an arginine residue at the P1 position of the activation site. At the cell surface, the activation of meprin *β* can be achieved by membrane-bound matriptase 2 (Jackle et al., 2015) or as a shed protein by soluble serine proteases, such as kallikrein 4/5 or pancreatic trypsin (Ohler et al., 2010). Of note, once activated at the plasma membrane, meprin *β* cannot be shed from the cell surface anymore (Wichert et al., 2017). Hence, meprin *β* occurs as a membrane-bound sheddase or soluble protease with access to different protein substrates.

Proteolysis of extracellular matrix and adhesion molecules is a very important factor in the context of cancer progression and metastasis. Meprin *β* is capable of cleaving off the prodomains of fibrillar collagens I and III, thereby inducing a collagen fibril assembly and deposition (Kronenberg et al., 2010; Broder et al., 2013). In this regard, meprin *β* has been associated with fibrotic conditions of the skin and in the lungs (Becker-Pauly et al., 2007; Biasin et al., 2014). On the other hand, meprin *β* is able to degrade collagen IV, an important component of the basal membrane, and thus may contribute to cancer cell metastasis (Kruse et al., 2004). Furthermore, meprin *β* has been shown to cleave different cell adhesion molecules. The expression of E-cadherin, a major adhesion molecule of the epithelium, was shown to be decreased compared to carcinoma and adenoma (Perl et al., 1998). Interestingly, E-cadherin is a substrate of meprin *β* (Huguenin et al., 2008), which could lead, in the case of dysregulation in the expression and functionality due to mutations, to tumor progression from adenoma to carcinoma. The adhesion molecule CD99 is overexpressed in many types of cancer, particularly in Ewing sarcoma and specific subtypes of leukemia (Manara et al., 2018; Pasello et al., 2018). CD99 is crucial for the transendothelial migration (TEM) of leukocytes promoting the final step of cell extravasation. The type I transmembrane protein is expressed on hematopoietic and endothelial cells (Jefferson et al., 2013; Bedau et al., 2017a; Bedau et al., 2017b). Being a substrate of meprin β, the cleavage of CD99 could influence tumor metastasis.

Another substrate of meprin *β* is the interleukin-6 receptor (IL-6R), which in its shed soluble form can induce a strong proinflammatory stimulus *via* the so-called IL-6 trans-signaling (Rose-John, 2012). Inflammatory processes and cancer progression are highly connected, mediated by cytokines like the IL-6R and other immunomodulatory molecules in the tumor microenvironment (Balkwill and Mantovani 2001; Fisher et al., 2014). It was observed that a selective blockage of IL-6 trans-signaling with the sgp130Fc protein (Olamkicept) had a suppressive effect on the tumorigenesis and metastasis of colorectal cancer (Schmidt et al., 2018; Schumacher and Rose-John 2019).

Meprin *β* has been associated with certain types of cancer, and its expression was observed in pancreatic and neuroendocrine tumors (Carr et al., 2013). Searching the BioMuta database (Dingerdissen et al., 2018), several single nucleotide variants (SNVs) of the Mep1b gene can be found in different cancer entities identified by multiple genomic studies, with the largest number identified in melanoma, uterine cancer, and lung cancer (Peters and Becker-Pauly, 2019). In this study, we characterized the melanoma-associated meprin *β* variants G45R and G89R with regard to cell surface expression, shedding activity, cell proliferation, and tumor cell invasion.

## Materials and Methods

### Chemicals

All chemicals were of analytical grade and obtained from Carl Roth GmbH + Co. KG, Merck KGaA, and Sigma-Aldrich Inc., and Thermo Fisher Scientific Inc., if not stated otherwise.

### Cells, Transfection, Plasmids, and Antibodies

ADAM10^−/−^; 17^−/−^ HEK293T cells (Riethmueller et al., 2016), deficient for the metalloproteases ADAM10 and ADAM17, were kindly provided by Björn Rabe, University of Kiel. HeLa and COS-7 cells were obtained from DSMZ GmbH (Braunschweig, Germany), and Ba/F3-gp130 cells (Gearing et al., 1994) were obtained from Immunex (Seattle, WA, United States). All cells were grown in DMEM (Dulbecco’s modified Eagle’s medium) and high glucose culture medium (Sigma-Aldrich) supplemented with 10% fetal calf serum (FCS), L-glutamine, and 1% penicillin and streptomycin (Thermo Fisher Scientific) and cultured at 37°C in 5% CO_2_ atmosphere and at 95% relative humidity. Ba/F3-gp130 cells were cultured using 10 ng/ml recombinant hyper-IL-6, which was expressed and purified as described previously (Fischer et al., 1997). Transient transfection was performed with polyethylenimine according to the manufacturer’s instructions. The following plasmids were used: human meprin *β* in the pcDNA4/TO-3x-Flag vector, human meprin *β* G45R in the pcDNA4/TO-3x-Flag vector, human meprin *β* G89R in the pcDNA4/TO-3x-Flag vector, human IL-6R, human CD99-Myc in pCMV6, APP695 in pCI-neo, and pcDNA3.1 as the empty vector control. The following antibodies were used: polyclonal anti–meprin *β* (generated against the ectodomain peptide CGMIQSSGDSADWQRVSQ, Pineda Antibody-Service, Berlin, Germany), monoclonal anti-IL-6R (4–11, generated against the D1 domain), monoclonal anti–glyceraldehyde-3-phosphate dehydrogenase (GAPDH) (2,118, 14C10; Cell Signaling Technology, Danvers, MA, United States), monoclonal anti-Flag (M2, F1804; MilliporeSigma), polyclonal anti-transferrin receptor (ab84036; Abcam, Cambridge, United Kingdom), monoclonal anti-Myc (9B11, 2,276; Cell Signaling Technology), phosphorylated signal transducer and activator of transcription 3 (pSTAT3) (9,131, Y705, Cell Signaling Technology, Danvers, MA, United States), STAT3 (9,139, 124H6, Cell Signaling Technology, Danvers, MA, United States), monoclonal anti–N-terminal APP (22C11; MilliporeSigma), polyclonal C-terminal anti-APP (CT15) and monoclonal anti–soluble APPα (6E10; Covance, Princeton, NJ, United States), and anti-PDI6 (ab11432; Abcam, Cambridge, United Kingdom).

### Generating Meprin *β* Variants G45R and G89R

Using the meprin *β* pcDNA4/TO construct with a C-terminal FLAG-tag as the template, the two variants G45R and G89R were generated. For exchanging single nucleotides, we applied appropriate primers (meprin *β* G45R: 5′-CAA​TGA​AGG​TTT​GAG​ACT​GGA​TCT​TTT​TGA​GGG-3′ and 5′-CCCT CAA​AAA​GAT​CCA​GTC​TCA​AAC​CTT​CAT​TG-3′ and meprin *β* G89R: 5′-GGAAATG AATGCTAAG CGA​GTT​ATC​CTC​AAT​GC-3′ and 5′-GCA​TTG​AGG​ATA​ACT​CGC​TTA​GCA​TTC​ATT​TCC-3′) and the QuickChange II XL Site-Directed Mutagenesis Kit (Agilent Technologies, Santa Clara, CA, United States) following the manufacturer’s instructions. To validate the correct single nucleotide exchanges of variants G45R and G89R, the cDNA of the Mep1b gene was sequenced by GATC Biotech, Konstanz, Germany.

### Cell Lysis, SDS-PAGE, and Western Blot Analysis

Transfected cells were harvested 48 h after transfection. For C-terminal fragment analysis, cells were treated with 1 μM γ-secretase inhibitor DAPT overnight prior to cell lysis. PBS-washed cells were lysed in 1% Triton X-100 and the protease inhibitor tablet with EDTA (Roche) in PBS. The protein concentration was determined using the BCA protein assay kit (Thermo Fisher Scientific) following the manufacturer’s instructions. Samples were heated in sample buffer including DTT for 10 min at 95°C. The total protein lysate (30 µg) was separated by SDS-PAGE and transferred onto PVDF or nitrocellulose membranes (GE Healthcare, Waukesha, WI, United States). Membranes were blocked with 5% dry milk or 3% BSA diluted in TBS for 1 h at room temperature, incubated overnight with the primary antibody in milk at 4°C, washed three times with TBS, and incubated with horseradish peroxidase–conjugated secondary antibody in TBS at room temperature. After further washes, membranes were developed with SuperSignal West Femto (Thermo Fisher Scientific) in a chemiluminescence detection system (LAS-3000; Fujifilm, Tokyo, Japan).

### Protein Deglycosylation

The total protein lysate (60 µg) of transfected ADAM10^−/−^; 17^−/−^ HEK293T cells was deglycosylated using PNGase F according to the manufacturer’s instructions (P0704; New England Biolabs, Ipswich, MA, United States).

### Cell Surface Biotinylation

ADAM10^−/−^; 17^−/−^ HEK293T cells were transiently transfected with meprin *β* variants or empty vectors and incubated for 24 h. Cells were washed twice with ice-cold PBS–CaCl_2_ and MgCl_2_ (CM) (CM: 0.1 mM CaCl_2_ and 1 mM MgCl_2_ in PBS) and treated with 1 mg/ml biotin solution (Sulfo-NHS-SS-Biotin; Thermo Fisher Scientific) in PBS-CM for 30 min at 4°C. The biotin solution was removed, and cells were incubated with quenching buffer (50 mM Tris-HCl in PBS-CM, pH 8) for 10 min at 4°C, washed three times with PBS-CM, and harvested. Subsequently, cells were lysed and the protein concentration was determined using the BCA assay kit following the instruction manual. For the purification of biotin-labeled proteins, streptavidin-coated magnetic beads (88,816, Thermo Fisher Scientific) were used according to the manufacturer’s instructions.

### Immunofluorescence Microscopy

The immunofluorescence staining of Cos-7 cells, which were seeded on coverslips, was performed 24 h after transfection as described previously (Peters et al., 2019). In brief, cells were washed three times with PBS and fixed with 4% (w/v) paraformaldehyde in PBS for 10 min. After cell permeabilization with 0.2% (w/v) saponin, cells were incubated with the primary antibody (anti-Flag 1:2000 and anti-PDI6 1:1000 in 1xPBS with 10% FCS) for 1 h, washed, and then incubated with the respective secondary antibody (Alexa Fluor 488 donkey anti-rabbit and Alexa Fluor 594 donkey anti-mouse, 1:300; Thermo Fisher Scientific). The excessive antibody was removed by five washes in 0.2% (w/v) saponin and PBS and two washes in double-distilled H_2_O. The coverslips were mounted onto slides with a mixture of 17% (w/v) Mowiol, 33% (v/v) glycerol, and 50 mg/ml 1,4-diazabicyclo[2.2.2]octane (MilliporeSigma) supplemented with 1 μg/ml DAPI for nuclear staining. Images were acquired on an Olympus FV 1000 confocal laser scanning microscope (Olympus, Hamburg, Germany).

### Quenched Fluorogenic Peptide Cleavage Assay

To quantify meprin *β* activity in cell lysates and at the cell surface, a highly specific fluorogenic peptide substrate for meprin *β* [7-methyloxycoumarin-4-yl (mca)–EDEDED–K-ε-2,4-dinitrophenyl (dnp), Genosphere Biotechnologies, Paris, France] was used in a final concentration of 50 µm (Jackle et al., 2015). For meprin *β* cell surface activity, transfected ADAM10^−/−^; 17^−/−^ HEK293T cells were washed three times in PBS and activity measurements were performed with 0.5 × 10^6^ cells in 48-well plates in a total volume of 300 µl. For cell lysate activity, cells were lysed using EDTA free lysis buffer, and a total amount of 50 µg protein was used for the activity assays. For trypsin activation, cells and lysates were incubated with 10 μg/ml of recombinant trypsin (Sigma-Aldrich) for 30 min at 37°C prior to measurement. Remaining cell suspensions were used for Western blot analyses. All activity assays were carried out in duplicates at 37°C. Proteolytic activity was measured as relative fluorescence units (RFUs) every 30 s for 120 min at an excitation of 405 nm and emission of 320 nm using the Tecan Infinite® F200 PRO plate reader (Tecan Trading AG). The activity was determined from the slope of the linear range of the curve normalized to the initial point of the measurement and presented as a bar graph.

### Ba/F3-gp130 STAT3 Phosphorylation Assay

The biological activity of the sIL-6R was analyzed as described previously (Sammel et al., 2019). In brief, after washing with PBS, Ba/F3-gp130 cells were incubated in serum-free medium for 2 h. Cells were incubated with an ultracentrifuged (186,000 x g for 2 h at 4°C), conditioned cell culture supernatant of ADAM10^−/−^; 17^−/−^ HEK293T cells co-transfected with meprin *β* variants and the IL-6R; 150 ng recombinant IL-6 was added. 150 ng hyper-IL-6 (Fischer et al., 1997; Schroers et al., 2005) served as positive control. Cells were incubated at 37°C and were shook for 15 min at 500 rpm followed by centrifugation at 1000 x g for 5 min at room temperature. After discarding the supernatant, cells were lysed in sample-buffer for 10 min at 95°C. Phosphorylated signal transducer and activator of transcription 3 (p-STAT3) levels were detected by Western blot analysis. STAT3 served as the control.

### Real Time Cell Analysis Invasion Assay

A real-time cell analysis (RTCA) system was used for testing the invasiveness of cells overexpressing meprin *β* and its variants G45R and G89R. RTCA (ACEA Bio, San Diego, CA, United States) is a method measuring the density-dependent variation of cell population growth using a sensitive micro-sensor system. The CIM-plate 16 used in this experiment is a two-chamber-well within a micro-sensor plate beneath a membrane with 8 µm pores. The top of the membrane was coated with a layer of collagen IV (Sigma-Aldrich C5533, 12 µg per well). The medium was measured to detect the background impedance. HeLa cells were transiently transfected with the meprin *β* variants, and 40.000 cells in a volume of 100 µl medium with 1% FCS were transferred into the upper chamber onto the layer of collagen IV. To prevent the cells from evaporating, the plate around the wells was filled with sterile PBS and covered with a lid. Pre-incubation of 1 h enabled all cells to adhere onto the layer of collagen IV before starting the RTCA experiment. RTCA was run for 24 h, measuring the cell index every 30 min at 37°C and 5% CO_2_. The cells passing through the layer of collagen IV touching the microelectrode increased the impedance. This was calculated automatically in the dimensionless cell index. The equation for the cell index is as follows: CI = (Z_i_–Z_0_)/15 Ω. Z_i_ is the individual impedance of each well to a certain time point and Z_o_ is the impedance of the time point at 15 min at the beginning. To be able to ignore a possible variability of cell numbers between the wells, a delta cell index was calculated and added. The formula for the delta cell index is as follows: DCI_ti_ = CI_ti_ + (DCI_reference_–CI_Delta_time_). Therefore, the delta cell index is a constant number for each well representing the difference between reference DCI and the cell index. For statistical analysis, the area under the curve (AUC) or the end-point number was used.

### Homology Modeling

The model of the membrane-bound meprin *β* dimer was built based on the crystal structure of human pro-meprin *β* (PDB ID: 4GWM) and a molecular model of the membrane-bound form, containing the EGF-like domains, the transmembrane helix, and the C-terminal tail (Arolas et al., 2012), using the SWISS-MODEL workspace (Waterhouse et al., 2018). Structure visualization was carried out using PyMOL (Schrödinger, New York, NY, United States).

### Statistical Analysis

All statistical analyses were performed using Prism 8 software (GraphPad Software, La Jolla, CA, United States) for a one-way ANOVA followed by Tukey’s post-test. Values are expressed as means ± sd. The null hypothesis was rejected at a value of *p* < 0.05.

## Results

### Localization and Cell Surface Expression of Meprin *β* Variants G45R and G89R

**FIGURE 1:**
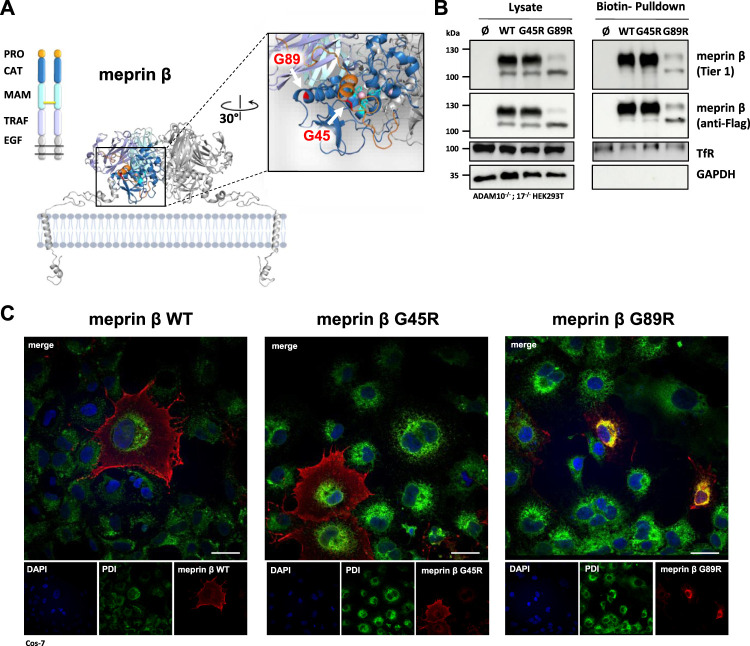
Expression and localization of G45R, G89R, and wild-type meprin *β*. **(A)** Domain composition (left) and the dimeric structure of the metalloprotease meprin *β* (right). The cartoon representation of membrane-bound meprin *β* based on the crystal structure of the ectodomain of human pro-meprin *β* (PDB: 4GWM), with one monomer colored according to the domain composition and the other one in light gray. The close-up view on the catalytic domain (blue) in the right panel shows the pro-peptide (orange) and the three active site histidine residues (cyan) that coordinate the zinc ion (pink). The positions of the two amino acid exchange variants G45R and G89R are highlighted in red. Pro-peptide, CAT: catalytic domain, MAM: meprin A5 protein tyrosine phosphatase *µ* domain, TRAF: tumor-necrosis-factor-receptor-associated factor domain, EGF: epidermal growth factor–like domain. The disulfide bridge between the MAM domains responsible for dimerization is indicated as a yellow bar. **(B)** Transfection of ADAM10^−/−^; 17^−/−^ HEK293T cells with the indicated meprin *β* variants. Cell surface proteins were labeled by primary amine biotinylation, pulled down with streptavidin sepharose beads, and analyzed *via* immunoblotting. GAPDH and transferrin receptor (TfR) served as the loading controls. **(C)** Immunofluorescence images of Cos-7 cells expressing the indicated meprin *β* variants (red), with the endoplasmic reticulum marker protein disulfide isomerase (PDI, green) and DAPI-stained nuclei (blue). Scale bars, 20 µm.

Based on the crystal structure of meprin *β,* we hypothesized a decrease in proteolytic activity for the G89R variant, having an additional arginine at the back of the catalytic domain at the interface to the TRAF domain, which may disturb proper folding (Figure 1A). The second meprin *β* variant G45R could insert a potential cleavage site into the propeptide, which likely promotes faster activation of the protease (Figure 1A). Since meprin *β* can be activated at the cell surface or in its soluble shed form, it was important to first investigate whether the cancer-associated variants G45R and G89R are correctly transported to the cell surface. Therefore, we performed cell surface biotinylation experiments of transfected HEK293T cells, deficient for ADAM10 and ADAM17, to prevent physiological ADAM-mediated shedding of meprin *β*. The biotin pulldown showed comparable cell surface levels for wild-type meprin *β* and the G45R variant, whereas clearly less levels of the meprin *β* variant G89R were detected at the cell surface (Figure 1B). However, it has to be considered that, on the one hand, the overall expression level of the meprin *β* variant G89R was clearly reduced in comparison to wild-type meprin *β* and G45R. On the other hand, the relative amount of the less glycosylated ER-form of meprin *β* detectable at about 100 kDa (Peters et al., 2019) was increased compared to the fully glycosylated form at about 130 kDa in the cell lysates of meprin *β* G89R (Supplementary Figure S1).

Within the biotinylated fractions of the meprin *β* variants, the majority of the detected meprin *β* corresponds to the higher molecular mass in the case of wild-type meprin *β* and its G45R variant, whereas only a similar trend was observed for meprin *β* G89R (Figure 1B). This suggests that the G89R mutation has a fold-impairing effect for meprin *β* that also affects its efficient N-glycosylation and transport to the cell surface.

The results were confirmed employing immunofluorescence microscopy of Cos-7 cells overexpressing the different meprin *β* variants (Figure 1C). Cos-7 cells were used instead of HEK293T cells due to their large size and better visibility of cellular compartments. While the G45R variant showed cell surface expression comparable to wild-type meprin *β*, the majority of meprin *β* G89R strongly co-localized with the endoplasmic reticulum marker protein disulfide isomerase (PDI). Overall, these results indicate that the G89R variant is, indeed, stuck on the secretory pathway probably due to impaired folding, causing a less efficient glycosylation.

### Activation and Proteolytic Activity of Meprin *β* Variants G45R and G89R Using a Specific Fluorogenic Peptide Substrate

**FIGURE 2:**
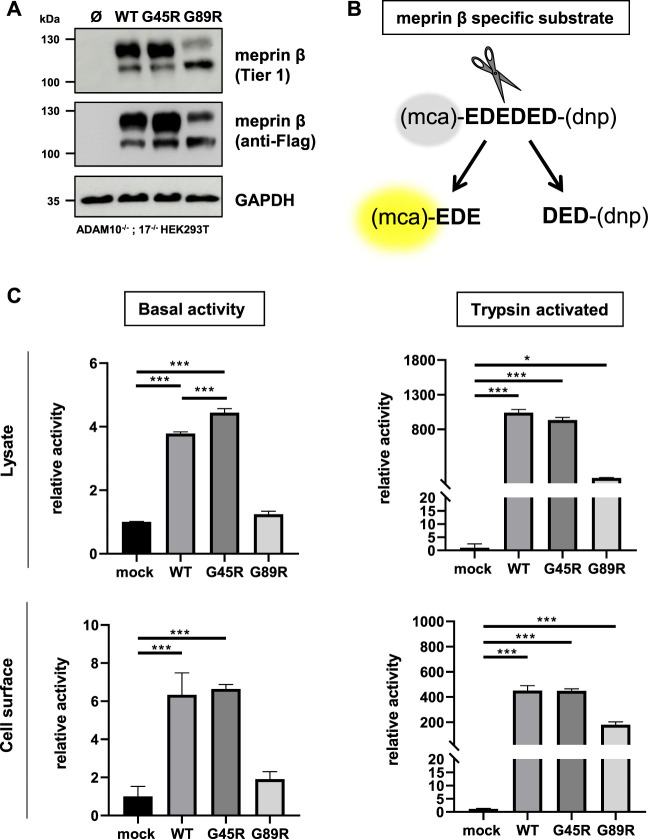
Proteolytic activity of G45R, G89R, and wild-type meprin *β* toward a fluorogenic peptide. ADAM10^−/−^; 17^−/−^ HEK293T cells were transiently transfected with a control plasmid (Ø) or one of the indicated meprin *β* variants. **(A)** Representative Western blot analysis of cell lysates. **(B)** Representation of the meprin *β*-specific quenched fluorogenic peptide used for activity assays. **(C)** Cell lysate and cell surface meprin *β* activity in the absence and presence of trypsin. Data are presented as means ± sd, and statistical analysis was assessed by the one-way ANOVA followed by Tukey’s post-test from three biological replicates. *p < 0.05, **p < 0.01, ***p < 0.001.

In order to investigate the functional consequences on the activation and proteolytic activity of the meprin *β* variants G45R and G89R, we initially performed activity assays in cell lysates and at the cell surface of transiently transfected ADAM10^−/−^; 17^−/−^ HEK293T cells (Figure 2A) using a meprin *β–*specific fluorogenic peptide (Figure 2B). Indeed, the meprin *β* G45R variant with a potential additional activation site did show significantly increased basal activity in cell lysates, while the cell surface activity was comparable to wild-type meprin *β* (Figure 2C). However, treating cell lysates and cells with the described pro-meprin *β* activator trypsin (Ohler et al., 2010; Jackle et al., 2015) did not result in increased proteolytic activity of meprin *β* G45R (Figure 2C). In the case of the G89R variant, the basal proteolytic activity in cell lysates and at the cell surface appeared to be very low (Figure 2C), which is likely also a result of the reduced expression rate. Nevertheless, the addition of trypsin showed that this meprin *β* variant can be activated (Figure 2C), indicating that the amino acid exchange of G89R does not lead to a complete loss of function mutation.

### The Proteolytic Activity of Meprin *β* G45R and G89R Toward IL-6R, CD99, and APP

In addition to the fluorogenic peptide-based activity assays, we, in a next step, analyzed the proteolytic activity of the meprin *β* variants G45R and G89R toward three known membrane-bound meprin *β* protein substrates, namely, IL-6R, CD99, and APP.

### IL-6R Shedding by Meprin *β* Variants

**FIGURE 3:**
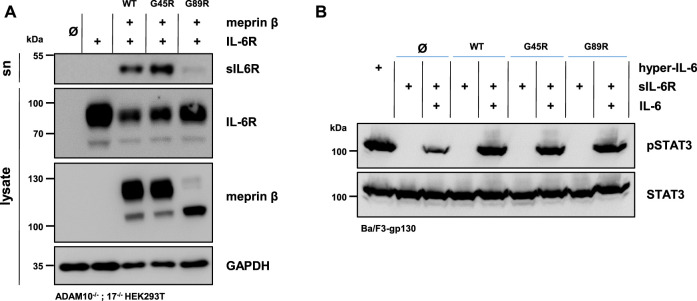
Meprin *β*-dependent shedding of the IL-6R and induced trans-signaling. **(A)** Immunoblots of ADAM10^−/−^; 17^−/−^ HEK293T cells transiently transfected with a control plasmid (Ø), IL-6R alone, and together with one of the indicated meprin *β* variants. Supernatants (sn) were ultracentrifuged and analyzed for sIL-6R. IL-6R and sIL-6R were detected with an antibody directed against the D1-domain of IL-6R and meprin *β* with an anti-Flag antibody. GAPDH served as the loading control. Of note, the amount of sIL-6R in the supernatant was increased upon the co-expression of G45R compared with meprin *β* WT. **(B)** Phosphorylation of STAT3 in Ba/F3-gp130 cells stably transfected with gp130. Cells were treated with ultracentrifuged supernatants from the experiments in **(A)** and the phosphorylation of STAT3 was analyzed in the presence and absence of 150 ng recombinant IL-6. The same amount of the fusion protein consisting of soluble IL-6R and IL-6 (hyper-IL-6) served as the positive control. Phosphorylation was detected with an antibody raised against phosphorylated STAT3 (pSTAT3). Total STAT3 protein served as the loading control. Western blot quantification of generated sIL-6R and pSTAT3 from three biologic replicates is presented in Supplementary Figure S2.

The soluble IL-6R plays an important role in cell proliferation and inflammation through trans-signaling (Rose-John, 2012). Not only ADAM10 and ADAM17 are capable of shedding the IL-6R (Mullberg et al., 1992) but also several other proteases, including meprin *β* (Arnold et al., 2017; Sammel et al., 2019). Similar to the single transfection of the three meprin *β* variants, the overall expression of meprin *β* G89R was also markedly reduced upon co-transfection with the IL-6R compared with the other two meprin *β* variants (Figure 3A, Supplementary Figures S2A,B). In line with the observed increased activity measured in the cell lysates (Figure 2C), co-transfection of meprin *β* G45R and the IL-6R in ADAM10^−/−^; 17^−/−^ HEK293T cells resulted in an increased release of the soluble IL-6R (sIL-6R) compared to wild-type meprin *β* (Figure 3A, Supplementary Figures S2A,B). Interestingly, meprin *β* G89R was also able to shed the IL-6R without further activation, albeit markedly less efficient than wild-type meprin *β* (Figures 2C, 3A; Supplementary Figures S2A,B). Normalizing the amount of generated sIL-6R to the IL-6R/meprin *β* levels of the cell lysate even suggests a similar proteolytic activity of meprin *β* G89R as wild-type meprin *β* (Supplementary Figure S2). However, this analysis might be rather misleading due to the obvious strongly reduced overall expression level of meprin *β* G89R. Nonetheless, the results suggest that the substrate binding to this meprin *β* variant has a folding stabilizing effect that renders the protease as proteolytically active.

In order to analyze the biological activity of the generated sIL-6R by the different meprin *β* variants, Ba/F3-gp130 cells were used. This murine pro-B-cell line (Ba/F3) is stably transfected with the signal transducing receptor gp130 and does not express the IL-6R endogenously (Taga and Kishimoto 1997). Therefore, these cells require either the sIL-6R and IL-6 or hyper-IL-6 (a chimeric fusion protein of the sIL-6R and IL-6) to induce trans-signaling by the JAK/STAT-pathway *via* phosphorylation of STAT3 (signal transducer and activator of transcription 3). In accordance with the immunoblot analyses, Ba/F3-gp130 cells were incubated with conditioned media from ADAM10^−/−^; 17^−/−^ HEK293T cells co-transfected with the IL-6R, and each of the three meprin *β* variants significantly induced STAT3 phosphorylation in dependence of the IL-6 in comparison to the mock control (Figure 3B, Supplementary Figure S2C).

### CD99 Shedding by Meprin *β* Variants

**FIGURE 4:**
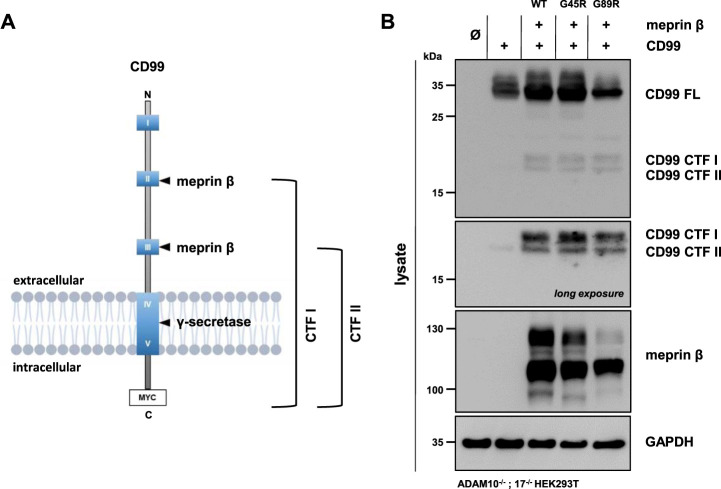
CD99 shedding by the meprin *β* variants G45R and G89R. **(A)** CD99 construct used in this experiment, highlighting the highly conserved acidic regions (HCRs) as blue boxes with roman numerals, the two known cleavage sites of meprin *β* and the C-terminal Myc-tag. **(B)** ADAM10^−/−^; 17^−/−^ HEK293T cells were transiently transfected with a control plasmid (Ø), CD99 alone, and together with one of the indicated meprin *β* variants. Cell lysates were analyzed by Western blotting using an anti-Flag antibody for meprin *β* and an anti-Myc antibody for CD99 detection (CD99 FL: CD99 full length; CD99 CTF I and CTF II: CD99 C-terminal fragments I and II). GAPDH served as the loading control. To determine the accumulation of γ-secretase–dependent cleavage products, the specific inhibitor DAPT was applied. Western blot quantification of generated CD99 CTFs from three biologic replicates is presented in Supplementary Figure S3.

Meprin *β* was shown to shed the adhesion molecule CD99, thereby promoting the transendothelial cell migration (TEM) of Lewis lung carcinoma (LLC) cells (Bedau et al., 2017a). The cleavage sites were identified in highly conserved acidic regions of CD99 (Bedau et al., 2017a). Comparable to JAM-A, ICAM-1, and L-selectin, CD99 controls the transendothelial migration of cells from the vessel lumen toward the interstitium for neutrophils, monocytes, and lymphocytes (Schenkel et al., 2002; Lou et al., 2007). Here, we investigated whether the meprin *β* variants G45R and G89R show altered cleavage efficiency of CD99. Therefore, ADAM10^−/−^; 17^−/−^ HEK293T cells were transfected with a C-terminally tagged CD99 alone and together with the meprin *β* variants. CD99 shedding was analyzed by the accumulation of C-terminal fragments CTF I and CTF II in cell lysates *via* immunoblotting, as described previously (Bedau et al., 2017a) (Figure 4, Supplementary Figure S3). To block further processing of the CTF II by the γ-secretase, the γ-secretase inhibitor N-[N-(3,5-difluorophenacetyl)-L- alanyl]-S-phenylglycine t-butyl ester (DAPT) was added to the transfected cells. As for the IL-6R, we observed that all variants of meprin *β* are capable of shedding CD99 and generating the fragments CTF I and CTF II (Figure 4B, Supplementary Figure S3). Similarly, the G45R variant showed a tendency of more efficient CD99 cleavage than wild-type meprin *β*, visualized by slightly increased levels of CTF I (Figure 4B, Supplementary Figure S3). In contrast, the G89R variant showed similar CD99 shedding capacity compared with wild-type meprin *β*, even without considering the normalization of the reduced expression of G89R relative to CD99 (Figure 4B, Supplementary Figure S3).

### APP Shedding by Meprin *β* Variants

**FIGURE 5:**
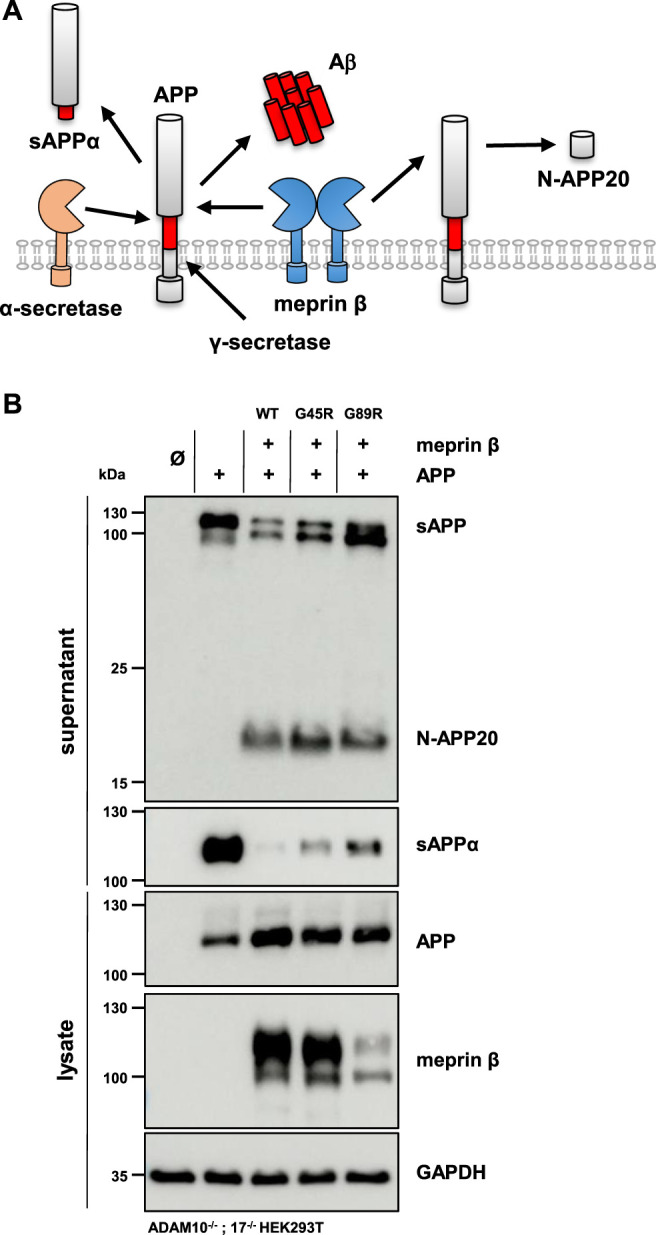
APP processing shedding by the meprin *β* variants G45R and G89R. **(A)** APP is cleaved by meprin *β* in two distinct ways. On the one hand, non-amyloidogenic N-APP fragments are produced, and on the other hand, meprin *β* acts as a *β*-secretase, competing with α-secretase cleavage at the cell surface. **(B)** ADAM10^−/−^; 17^−/−^ HEK293T cells were transiently transfected with a control plasmid (Ø), APP alone, and together with one of the indicated meprin *β* variants. Cell lysates and supernatants were analyzed by immunoblotting using an anti-Flag antibody for meprin *β* and specific APP antibodies (N-APP: 22C11, sAPPα: 6E10, and total APP in lysates: CT15). GAPDH served as the loading control. Western blot quantification of generated N-APP and sAPPα from three biologic replicates is presented in Supplementary Figure S4.

The altered glycosylation pattern of the meprin *β* variant G89R in cell lysates and biotinylated cell surface fractions together with its significantly reduced cell surface localization as judged by immunofluorescence microscopy has been similarly described for the meprin *β* variant D204A (Arnold et al., 2015). This mutation likewise results in the expression of a less glycosylated meprin *β* ER-form and shows only little cell surface activity compared to wild-type meprin *β*. However, it has a very high *β*-secretase activity toward the well-described meprin *β* substrate, amyloid precursor protein (APP), demonstrated by increased Aβ peptide generation (Arnold et al., 2015). Of note, APP processing by meprin *β*, either at the *β*-secretase site or at the N-terminus releasing N-APP fragments, has been observed to take place at the cell surface and even on the secretory pathway (Figure 5A) (Schonherr et al., 2016; Scharfenberg et al., 2019). Additionally, at least meprin *β* activity in transiently transfected HEK293 cells correlates negatively with sAPPα levels (Armbrust et al., 2021). The observed proteolytic activity of the meprin *β* G89R variant towards CD99 and the IL-6R suggests substrate binding and processing already on the secretory pathway. Therefore, APP represented an ideal substrate to verify this hypothesis. In order to do so, ADAM10^−/−^; 17^−/−^ HEK293T cells were transfected with APP alone or together with the meprin *β* variants. Even though these cells are deficient for the two α-secretases ADAM10 and ADAM17, APP processing at the α-secretase cleavage site is observed, most likely by a compensatory endogenous alternative α-secretase like, e.g., ADAM9 (Asai et al., 2003). However, co-expression of wild-type meprin *β* and its variants resulted in the expected meprin *β*-dependent APP cleavage pattern (Figure 5B), with a comparable increase of N-APP20 fragments in the supernatant and decreased sAPPα levels (Figure 5B). Similarly, as observed for meprin *β* D204A, APP processing by meprin *β* G89R resulted in N-APP20 cleavage. However, in the case of meprin *β* G89R, sAPPα cleavage was higher compared to meprin *β* wild-type and G45R (Figure 5B). Similarly, as observed for CD99 shedding, the normalization of meprin *β* G45R proteolysis to overall expression levels would suggest that meprin *β* G45R is even more active than wild-type meprin *β* (Supplementary Figure S4B). Taken together, these findings suggest that meprin *β* G89R is, indeed, mainly proteolytically active on the secretory pathway.

### Meprin *β* G89R Diminishes the Invasion of Cancer Cells Through Collagen IV

**FIGURE 6:**
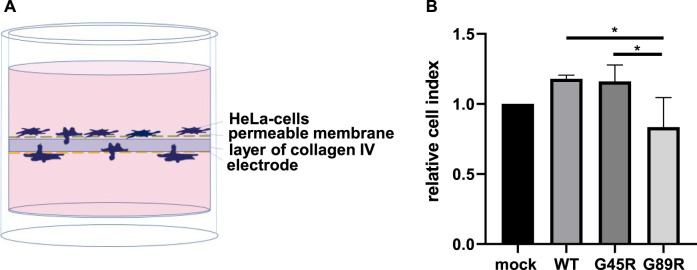
Cancer cell invasion assay through collagen IV matrix. **(A)** HeLa cells were transfected and seeded into the upper chamber of the well, and the impedance of the electrode located at the bottom of the well was measured for 24 h. An increase in impedance was equal to cell invasion through the layer of collagen IV. **(B)** Quantification of the results demonstrates that the expression of G89R reduced the invasiveness significantly. Values of the different meprin *β* variants were normalized to values of mock-transfected cells, which were set as 1. Data are presented as means ± sd, and statistical analysis was assessed by the one-way ANOVA followed by Tukey’s post-test from three biologic replicates. *p < 0.05, **p < 0.01, ***p < 0.001.

Since invasiveness is an important characteristic of cancer cells, we investigated whether meprin *β* variants identified in certain tumors have an influence on this process. Collagen IV is a major component of the basal lamina and thus part of an important barrier separating the epithelia from underlying tissues. It has been observed that meprin *β* is capable of cleaving collagen IV, which may contribute to cancer cell invasiveness (Kruse et al., 2004). Therefore, we analyzed the invasive mobility of cells passing through a semipermeable membrane coated with collagen IV (Figure 6). HeLa cells transfected with meprin *β* WT, G45R, and G89R or mock as negative control were transferred into wells with two chambers, divided by a layer of collagen IV (Figure 6A). Cells passing through that layer changed the impedance of the electrode placed on the bottom side. Therefore, the change in impedance was equal to the number of cells, which had passed through the layer of collagen IV. In contrast to the cell surface–expressed meprin *β* WT and G45R, the expression of the meprin *β* G89R variant significantly diminished the invasiveness of HeLa cells (Figure 6B).

## Discussion

The dysregulation of proteolytic enzymes is often associated with tumor progression and metastasis, for instance, by matrix metalloproteases (MMP) degrading extracellular matrix (Winer et al., 2018). This is often caused by an increased expression of proteases by certain cancers, which may induce proliferation or mobility of these cells. Latest genomic approaches revealed that malignant hyperplasia exhibits a number of different driver or bystander mutations and several of these were identified in protease genes. Here, we investigated two cancer-associated variants of the metalloprotease meprin *β*, namely, G45R and G89R, both leading to an exchange of the amino acid glycine to arginine. We functionally characterized these variants in biochemical and cell biological approaches to determine possible consequences for cancer cell invasiveness.

Based on the crystal structure of meprin *β* (Arolas et al., 2012), we decided to investigate the biochemical properties and enzymatic functions of the meprin *β* variants G45R and G89R. The G45R mutation is located within the propeptide of meprin *β*. Knowing that the cleavage of the propeptide occurs C-terminal of the arginine at position 61 (Jackle et al., 2015), the insertion of another arginine in close proximity could lead to an enhanced activation of meprin *β*. For the G89R variant, we presumed that the insertion of arginine with a big positively charged sidechain into the very tight area between the catalytic domain and the TRAF domain would have a negative effect on the proteolytic activity due to impaired folding of the protein.

Indeed, we observed decreased levels of fully glycosylated G89R in cell lysates and at the plasma membrane compared to wild-type meprin *β*. Along the same lines, we detected higher amounts of the less glycosylated ER-form of meprin *β* in the case of the G89R variant. This indicates that the G89R variant was partially stuck on the secretory pathway, which could be confirmed by the immunofluorescence analysis where an ER-marker (PDI) showed strong co-localization with G89R. This observation was further supported by a markedly reduced proteolytic activity of this meprin *β* variant on the plasma membrane of living cells. Nonetheless, considering the protein substrate analyses including APP processing, it turned out that substrate binding on the secretory pathway seems to have a folding stabilizing effect, which enables G89R activation and, therefore, proteolytic activity toward substrates before reaching the cell surface.

Analyzing the meprin *β* G45R variant, we expected the protease to be faster activated due to the inserted arginine in position 45, representing a potential additional activation site, besides the arginine in position 61 that is cleaved by pancreatic trypsin and matriptase 2 (Ohler et al., 2010; Jackle et al., 2015). This was, indeed, true for the basal activity; however, we did not observe increased proteolytic activity on cells expressing the G45R variant when incubated with trypsin. In studies investigating a meprin *β* G32R variant, the additional arginine, indeed, resulted in increased cell surface activity of meprin *β* (Schäffler et al., 2019). Analyzing the proteolytic processing of protein substrates, G45R showed a tendency of increased activity compared to wild-type meprin *β*. One explanation why G45R is less prone to an increased activation than G32R could be the position of the mutation within the propeptide. In pro-meprin *β*, the propeptide interacts *via* two salt bridges on the prime site (D30-R146 and D34-R146) and two on the non-prime site (R54-E137 and D56-R131) with the catalytic domain (Arolas et al., 2012). Therefore, it is possible that G32R has a more destabilizing impact by disrupting the prime site interaction than G45R, which is located in the middle of the two important interaction sites. Consequently, G32R leads to more pronounced meprin *β* activity at the cell surface, while the effect of G45R is rather moderate.

IL-6 classic- and trans-signaling are important mechanisms in immunomodulation, promoting immune cell differentiation and proliferation (Rose-John, 2012). For trans-signaling cleavage of the IL-6 receptor by ectodomain sheddases, such as ADAM17 or meprin *β*, it is mandatory to act on cells that do not express the receptor endogenously (Arnold et al., 2017; Sammel et al., 2019). The pro-B-cell line Ba/F3-gp130 is dependent on IL-6 trans-signaling as an inducer of proliferation (Sammel et al., 2019). In this study, we could show that the IL-6R cleavage was increased by the meprin *β* variant G45R in comparison to the wild-type enzyme, which could influence cancer and immune cell proliferation.

The meprin *β* substrate CD99 was observed to promote cancer cell extravasation (Bedau et al., 2017a; Bedau et al., 2017b). However, whether the persistent cleavage by the G45R and G89R variants as shown in this study has influence on cell migration in melanoma has to be further demonstrated (Wilkerson et al., 2006).

Natural tissue barriers such as the basal membrane are important factors for tumor progression and tumor metastasis (Tanjore and Kalluri 2006). Meprin *β* has been shown to hydrolyze collagen IV (Kruse et al., 2004), a major component of the basement membrane. Therefore, we investigated whether one of the protease variants would have an impact on the invasiveness of tumor cells *in vitro.* Testing the invasiveness of Hela cells expressing the different variants of meprin *β* revealed that meprin *β* in its active form on the cell surface (variant G45R und WT) is more capable of passing through a layer of collagen IV. Although the difference was not huge, an alternation of the proteolytic activity of meprin *β* likely affects the invasiveness of tumor cells. This result was in line with the results of the G32R variant (Schäffler et al., 2019), thus indicating a possible pro-metastatic function of meprin *β*.

Taken together, here we characterized biochemically and in cell-based assays the two annotated cancer-associated meprin *β* amino exchange variants G45R and G89R, indicating controversial functions on cancer progression. On the one hand, we identified potentially cancer-promoting functions of G45R, such as increased proteolytic activity at the cell surface. On the other hand, G89R showed overall reduced expression levels and was mainly active on the secretory pathway and only partially reached the plasma membrane. This rather indicates antitumorogenic properties particularly for meprin *β* substrates, which can be processed solely at the plasma membrane or substrates of the extracellular matrix, as shown by the cell invasion assay. Hence, future studies will show if the characterized meprin *β* variants are bystanders or driver mutations in melanoma.

## Data Availability

The original contributions presented in the study are included in the article/Supplementary Material; further inquiries can be directed to the corresponding author.
