# Supplementary material for: Characterization of the Cancer-Associated Meprin Βeta Variants G45R and G89R
Source: Front Mol Biosci. 2021 Oct 6;8:702341. doi: 10.3389/fmolb.2021.702341 (PMC8526939; doi:10.3389/fmolb.2021.702341)
Supplement: Supplementary file 1 [file DataSheet1.pdf]

Supplementary Figure 1

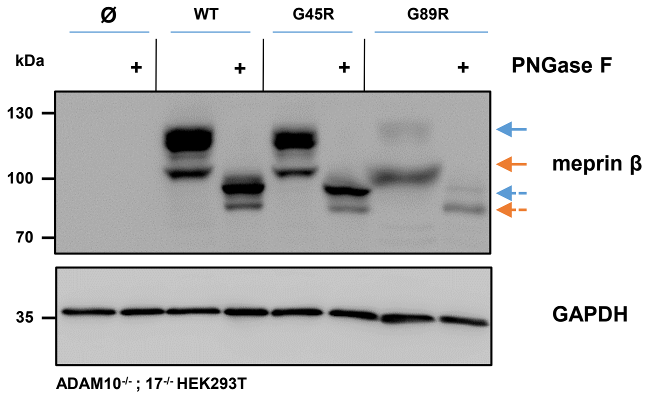

Supplementary Figure S1. Deglycosylation of wild-type meprin β and its variants G45R and G89R. ADAM10$^{-/-}$; 17$^{-/-}$ HEK293T cells were transiently transfected with a control plasmid (Ø) or with one of the indicated meprin β variants [wild-type (WT), G45R or G89R]. Protein lysates were treated with PNGase F and compared with untreated lysates by immunoblotting using an anti-Flag antibody for meprin β detection, GAPDH served as loading control. The fully glycosylated meprin β at about 130 kDa and the less glycosylated 100 kDa form of meprin β treated samples are marked with a blue and orange arrow, respectively. Dashed arrows highlight the deglycosylated meprin β.

Supplementary Figure 2

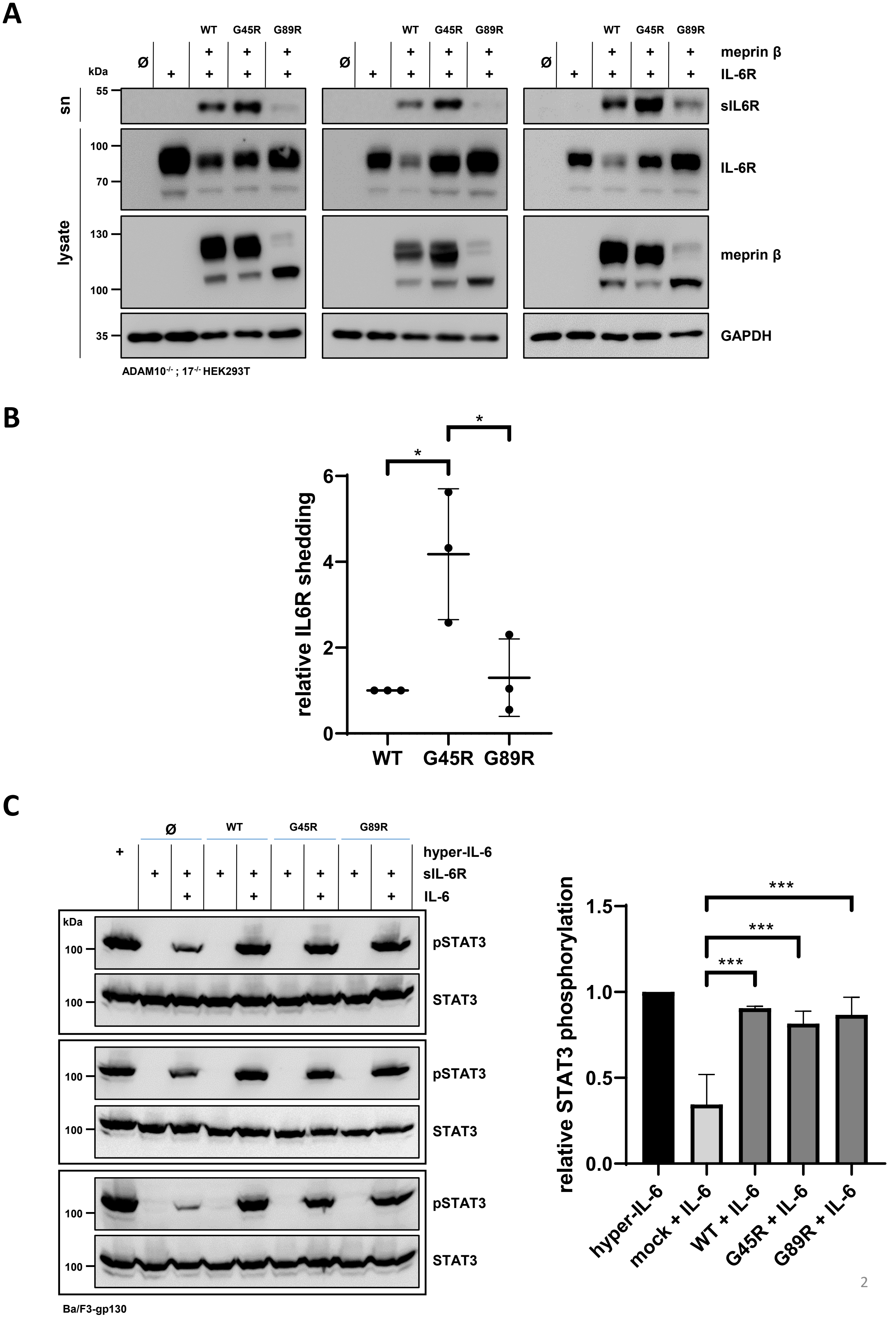

Supplementary Figure S2. Biological replicates of IL-6R shedding by wild-type meprin β and its variants G45R and G89R and induced trans-signaling.

(A) Three biologic replicates of ADAM10$^{-/-}$; 17$^{-/-}$ HEK293T cells transiently transfected with a control plasmid (Ø), IL-6R alone and together with one of the indicated meprin β variants. Supernatants (sn) were ultracentrifuged and analyzed for sIL-6R. IL-6R and sIL-6R were detected with an antibody directed against the D1-domain of IL-6R and meprin β with an anti-Flag antibody. GAPDH served as loading control. **(B)** Quantification of the three individual experiments shown in A. The relative amount of shed IL-6R was determined as the ratio of soluble IL-6R (sIL-6R) in the supernatant relative to the normalized expression level of IL-6R and meprin β in the cell lysates. Data are presented as means ± sd, and statistical analysis was assessed by 1-way ANOVA followed by Tukey's posttest. *P < 0.05, **P < 0.01, ***P < 0.001. **(C)** Phosphorylation of STAT3 in Ba/F3-gp130 cells stably transfected with gp130. Cells were treated with ultracentrifuged supernatants from the experiments in (A) and phosphorylation of STAT3 was analyzed in presence and absence of 150ng recombinant IL-6. The same amount of the fusion protein consisting of soluble IL-6R and IL-6 (hyper-IL-6) served as positive control. Phosphorylation was detected with an antibody raised against phosphorylated STAT3 (pSTAT3). Total STAT3 protein served as loading control. **(D)** Quantification of the relative amount of phosphorylated STAT3 (pSTAT3) of the three individual experiments shown in C. Data are presented as means ± sd, and statistical analysis was assessed by 1-way ANOVA followed by Tukey's posttest. *P < 0.05, **P < 0.01, ***P < 0.001.

Supplementary Figure 3

A

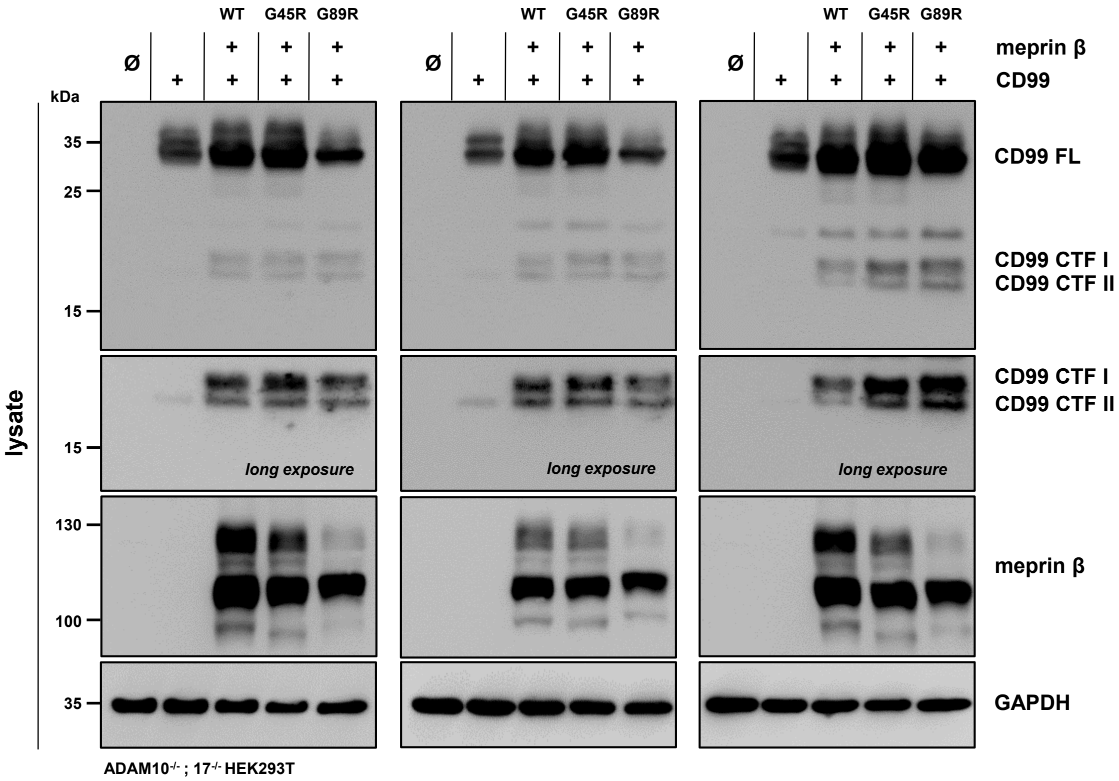

B

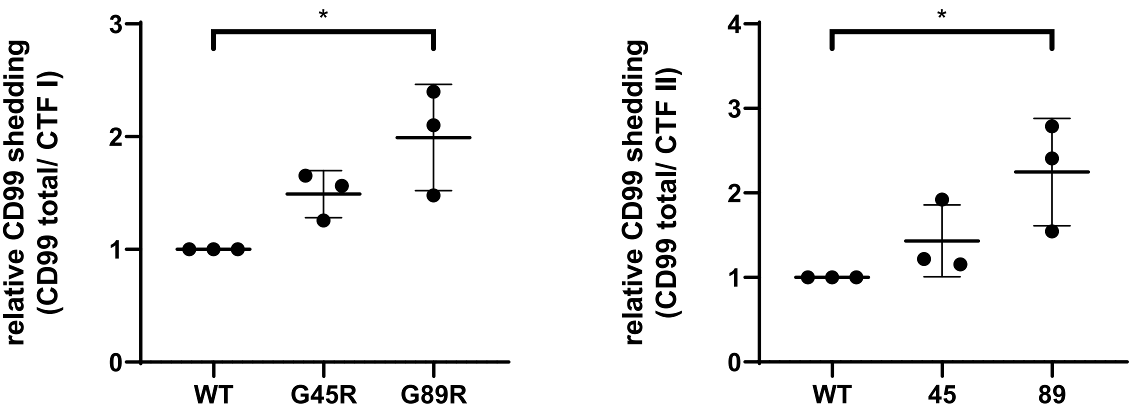

Supplementary Figure S3. Biological replicates of CD99 shedding by wild-type meprin β and its variants G45R and G89R.

(A) Three biologic replicates of ADAM10$^{-/-}$; 17$^{-/-}$ HEK293T cells transiently transfected with a control plasmid (Ø), CD99 alone and together with one of the indicated meprin β variants. Cell lysates were analyzed by immunoblotting using an anti-Flag antibody for meprin β and an anti-Myc antibody for CD99 detection (CD99 FL: CD99 full length, CD99 CTF I and CTF II: CD99 C-terminal fragments I and II). GAPDH served as loading control. To determine the accumulation of γ-secretase dependent cleavage products, the specific inhibitor DAPT was applied. **(B)** Quantification of the three individual experiments shown in A. The relative amount of shed CD99 was determined as the ratio of CTF I (left panel) and CTF II (right panel) relative to the total amount of CD99 (CD99 total) normalized to the overall expression level of CD99/ meprin β in the cell lysates. Data are presented as means ± sd, and statistical analysis was assessed by 1-way ANOVA followed by Tukey's posttest. *$P < 0.05$, **$P < 0.01$, ***$P < 0.001$.

Supplementary Figure 4

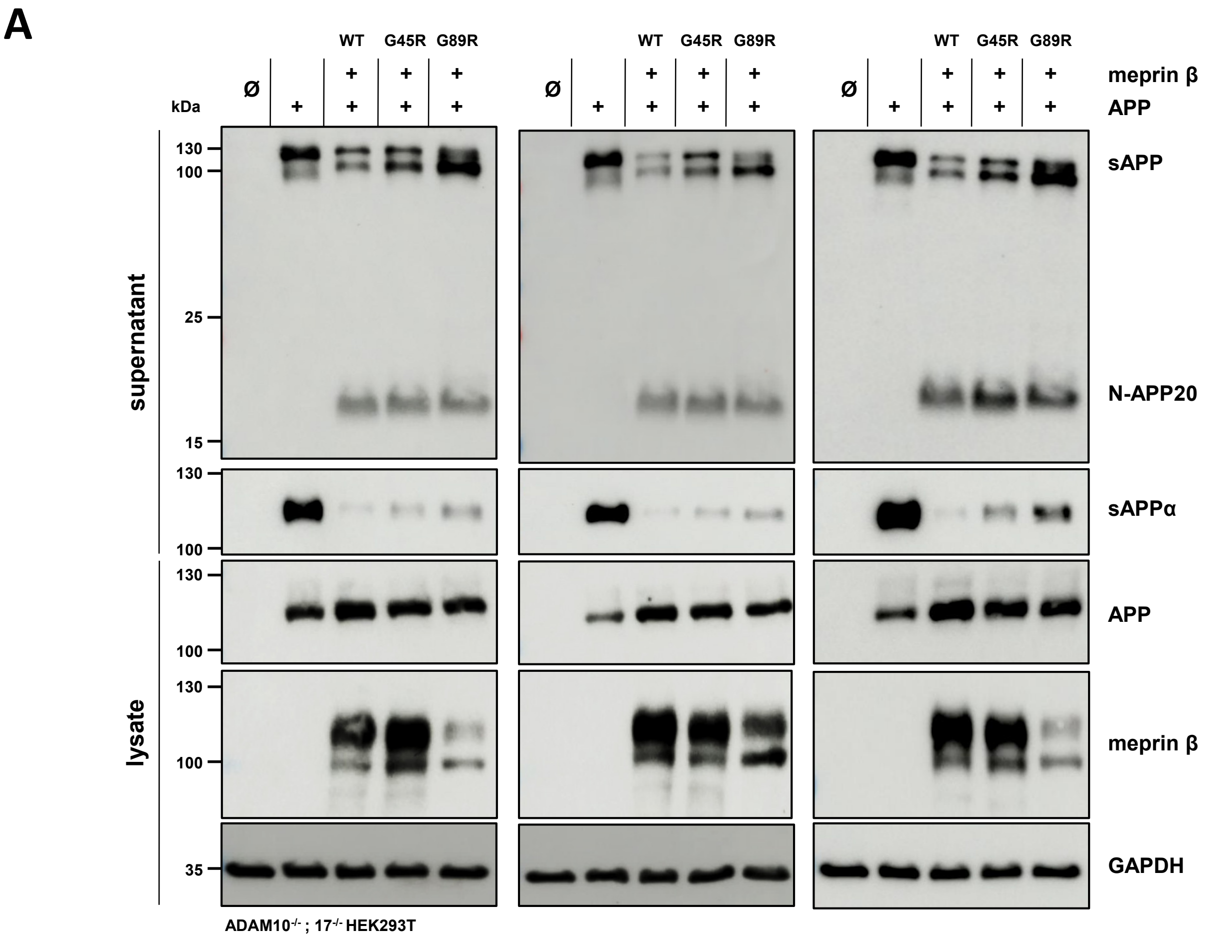

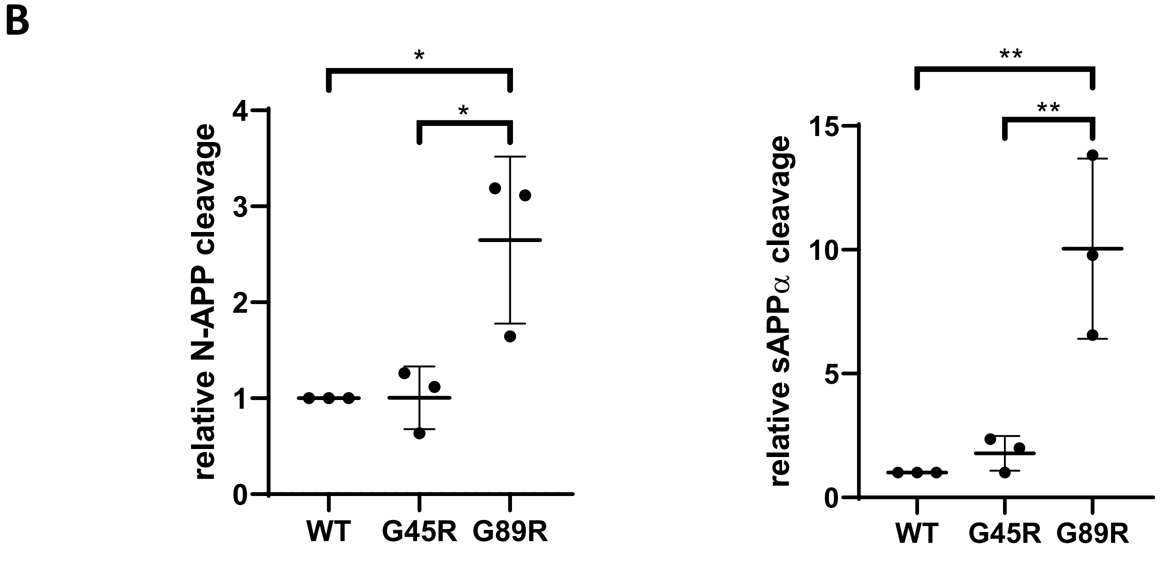

Supplementary Figure S4. Biological replicates of APP shedding by wild-type meprin β and its variants G45R and G89R.

(A) Three biologic replicates of ADAM10$^{-/-}$; 17$^{-/-}$ HEK293T cells transiently transfected with a control plasmid (∅), APP alone and together with one of the indicated meprin β variants. Cell lysates and supernatants were analyzed by immunoblotting using an anti-Flag antibody for meprin β and specific APP antibodies (N-APP: 22C11, sAPPα: 6E10, total APP in lysate: CT15). GAPDH served as loading control. **(B)** Quantification of the three individual experiments shown in A. The relative amount of N-APP (left panel) and sAPPα (right panel) cleavage in the supernatant was determined relative to the normalized expression level of APP and meprin β in the cell lysates. Data are presented as means ± sd, and statistical analysis was assessed by 1-way ANOVA followed by Tukey's posttest. *$P < 0.05$, **$P < 0.01$, ***$P < 0.001$.
